# Elevated Levels of Cerebrospinal Fluid and Plasma Interleukin-37 in Patients with Guillain-Barré Syndrome

**DOI:** 10.1155/2013/639712

**Published:** 2013-09-19

**Authors:** Cong Li, Pingwei Zhao, Xiguang Sun, Yuanyuan Che, Yanfang Jiang

**Affiliations:** Key Laboratory of Zoonosis Research, Ministry of Education, Institute of Zoonosis, The Second Part of First Hospital, Jilin University, Changchun 130032, China

## Abstract

*Aims*. Interleukin-37 (IL-37) is an anti-inflammatory cytokine. This study aims to investigate the concentrations of plasma and cerebrospinal fluid (CSF) IL-37 in patients with Guillain-Barré Syndrome (GBS). *Methods*. The levels of plasma and CSF IL-37, IL-17A, IFN-**γ**, and TNF-**α** in 25 GBS patients and 20 healthy controls (HC) were determined by enzyme-linked immunoabsorbent assay and flow cytometric bead array assay, respectively. The values of clinical parameters in the patients were also measured. *Results*. The concentrations of plasma IL-37, IL-17A, IFN-**γ**, and TNF-**α** and CSF IL-37 and IL-17A in patients at the acute phase of GBS were significantly higher than those in the HC. The levels of plasma IL-37, IL-17A, IFN-**γ**, and TNF-**α** were positively correlated in those patients, and the levels of CSF IL-37 and IL-17A as well as the levels of plasma TNF-**α** were correlated positively with the GBS disability scale scores (GDSs) in those patients. Treatment with intravenous immunoglobulin significantly reduced the levels of plasma IL-37, IL-17A, IFN-**γ**, and TNF-**α** in the drug-responding patients. *Conclusions*. Our findings indicate higher levels of plasma and CSF IL-37 and IL-17A and other proinflammatory cytokines in patients with GBS.

## 1. Introduction

Guillain-Barré syndrome (GBS) is an autoimmune disease characterized by autoimmunity against the peripheral nerves, leading to an acute polyneuropathy [1]. The incidence of GBS is 0.6–4.0 per 100,000 worldwide, and GBS preferably affects men [2]. The main clinical features of GBS are progressive, symmetrical muscle weakness associated with depressed or absent deep tendon reflexes [3]. The weakness frequently involves respiratory muscles, rendering patients respirator-dependent [4]. Typically, patients with GBS usually display albuminocytological dissociation in the cerebrospinal fluid (CSF) with abnormally increased levels of proteins but without increased numbers of cells during the first week of the acute phase of GBS [5]. During the pathogenic process of GBS, patients commonly show blood-nerve barrier (BNB) breakdown, cytokine leakage, and activated T-cell and macrophage infiltration in the peripheral nervous system (PNS) [6]. Currently, the etiology of GBS is unclear, although many factors such as bacterial or viral infections, vaccinations, and host susceptibility may contribute to the development of GBS [7]. However, how proinflammatory and anti-inflammatory cytokines are associated with the development of GBS has not been clarified.

A previous study has suggested that TNF-*α* can interfere with myelin protein and glycolipid synthesis and may be involved in the immune-mediated demyelination and axonal damage of peripheral nerves during the pathogenesis of GBS [8]. IFN-*γ* and IL-17 produced by proinflammatory Th1 and Th17 cells are important for the pathogenesis of GBS [9–11]. Given that Chinese patients are living in a unique environment, it is unclear how these proinflammatory cytokines regulate the pathogenesis of GBS in Chinese patients.

Interleukin-37 (IL-37), a member of the IL-1 cytokine family, is produced by various types of cells including NK cells, monocytes, activated B cells, and keratinocytes [12]. It includes five different splice variants of a–e, which are expressed in the different tissues, respectively [13]. IL-37a, b, and d are recognized as the functional forms of IL-37 [12]. The IL-37b has been identified as a natural suppressor of innate inflammatory and immune responses [14]. It is highly expressed in inflammatory tissues to inhibit the excessive inflammatory response [15]. However, there is no information about whether the levels of IL-37 in the CFS and peripheral blood alter during the pathogenic process of GBS and how the changes in the levels of CSF and plasma IL-37 are associated with disease activity in GBS patients. Currently, GBS patients are usually treated with intravenous immunoglobulin (IV-Ig) and plasma exchange, and the IV-Ig is preferred in China [4]. However, it is unclear whether the standard therapy can modulate the levels of CSF and plasma IL-37 in GBS patients.

In this study, we examined the levels of CSF and plasma IL-37, IL-17A, IFN-*γ*, and TNF-*α* in 25 patients with new onset GBS before and after the standard therapy as well as 20 age- and gender-matched healthy controls. We explored the potential association of the levels of CSF and plasma cytokines with disease activity in GBS patients.

## 2. Materials and Methods

### 2.1. Patients

Twenty-five patients with new onset GBS were recruited sequentially at the Neurology Department, and 20 age- and sex-matched healthy volunteers were recruited at the Physical Examination Center of the Second Part of the First Hospital of Jilin University from March 2012 to May 2013. Individual patients with GBS were diagnosed, according to the international diagnostic criteria [16]. The diagnosis of acute GBS was based on the following criteria: an acute progressive symmetrical weakness of the extremities with areflexia or hyporeflexia, albuminocytological dissociation in the CSF, and demyelinating/axonal neuropathy by electrophysiology. Albuminocytological dissociation was defined as abnormal levels of proteins but a total cell count of ≤10/mm^3^ in CSF. Individual patients were excluded if she/he had a history of autoimmune diseases, such as multiple sclerosis (MS), rheumatoid arthritis (RA), inflammatory bowel disease (IBD), and type 1 diabetes (T1D), or chronic inflammatory diseases, such as metabolic syndrome, type 2 diabetes, chronic cardiovascular disease, and malignancy, or a recent infection or if he/she was a heavy smoker.

The disease severity of individual patients with GBS was evaluated by experienced neurologists using the GBS disability scale scores (GDSs), a widely accepted scoring system to evaluate the functional status of GBS patients [17]. Briefly, the GBS at grade 0: normal neurological status; grade 1: minor symptoms, able to run; grade 2: limb weakness, able to walk 5 m unaided; grade 3: able to walk 5 m only with aid; grade 4: chair or bed bound; grade 5: requiring assisted ventilation; and grade 6: death. Written informed consent was obtained from individual participants, and the experimental protocol was approved by the Ethical Committee of the First Hospital of Jilin University. Their demographic and clinical characteristics are summarized in Table 1.

### 2.2. Specimen Collection and Preparation

Fasting blood and CSF samples of individual participants were collected within 48 h after admission. The CSF samples were donated by those healthy controls, which was approved by the Ethical Committee of the First Hospital of Jilin University. Additional blood samples were collected from individual patients at the recovery phase (at the fifth week after treatment). The blood and CSF samples were subjected to centrifugation, and the resulting plasma and CSF supernatants were stored at −80°C. The numbers of blood mononuclear cells in the CSF were counted in a blinded manner.

### 2.3. Treatment and Evaluation

Individual patients were treated intravenously with 0.4 g/kg/d of immunoglobulin daily for 5 consecutive days. Patients at the recovery phase with a GBS score of at least 1 less than that at the acute phase were considered as drug responders, while other patients were defined as the drug nonresponders.

### 2.4. Enzyme-Linked Immunosorbent Assay (ELISA) for the Measurement of IL-37

The levels of plasma and CSF IL-37 in the HC and GBS patients were determined using a commercially available human IL-37 ELISA kit (AdipoGen, Switzerland), according to the manufacturers' instruction. Briefly, individual plasma and CSF samples were diluted at 1 : 1 and tested in triplicate by ELISA. The concentrations of plasma and CSF IL-37 of individual samples were determined using the standard curve established using the recombinant IL-37 provided. The limitation of detection for human IL-37 was 10 pg/mL.

### 2.5. Cytometric Bead Arrays of Serum and CSF Cytokines

The levels of plasma and CSF IL-17A, IFN-*γ*, and TNF-*α* in the HC and GBS patients were determined by cytometric bead array (CBA) [18], according to the manufacturer's protocol (BD Biosciences, San Jose, CA, USA). Briefly, individual samples (25 *μ*L/tube) were tested in duplicate for the analysis of cytokines, as described previously [19]. The concentrations of plasma and CSF cytokines were quantified by flow cytometry analysis using the CellQuest Pro and CBA software (Becton Dickinson) on a FACSCalibur cytometer (BD Biosciences).

### 2.6. Statistical Analysis

Data are present as median and range, except when specified. The difference between the groups was analyzed by Mann-Whitney *U* test and Wilcoxon signed-rank test using SPSS 18.0 software for unpaired and paired comparisons, respectively. The relationship between the variables was evaluated using the Spearman rank correlation test. A two-side *P* value of <0.05 was considered statistically significant.

## 3. Results

### 3.1. Demographic and Clinical Characteristics of Study Subjects

Twenty-five GBS patients and 20 age- and gender-matched healthy controls (HC) were recruited. Their demographic and clinical characteristics are summarized in Table 1. There was not a significant difference in the distribution of age and gender between the GBS patients and the HC. While there was not a significant difference in the values of WBC in the CFS and the concentrations of plasma albumin between the GBS patients and the HC, the concentrations of CSF albumin in the patients were significantly higher than that in the HC (*P* < 0.05). As a result, the percentages of the concentrations of CSF albumin in plasma albumin in the patients were significantly higher than that in the HC (*P* < 0.05). Clearly, these patients had abnormally higher levels of CSF albumin but normal range of WBC counts, indicative of albuminocytological dissociation. Total numbers of white blood cells in the GBS patients were significantly higher than the HC, but the numbers of lymphocytes were similar between the GBS patients and the HC.

### 3.2. Higher Levels of Plasma and CSF IL-37 and IL-17A Are Detected in Patients with New Onset GBS

**Figure 1:**
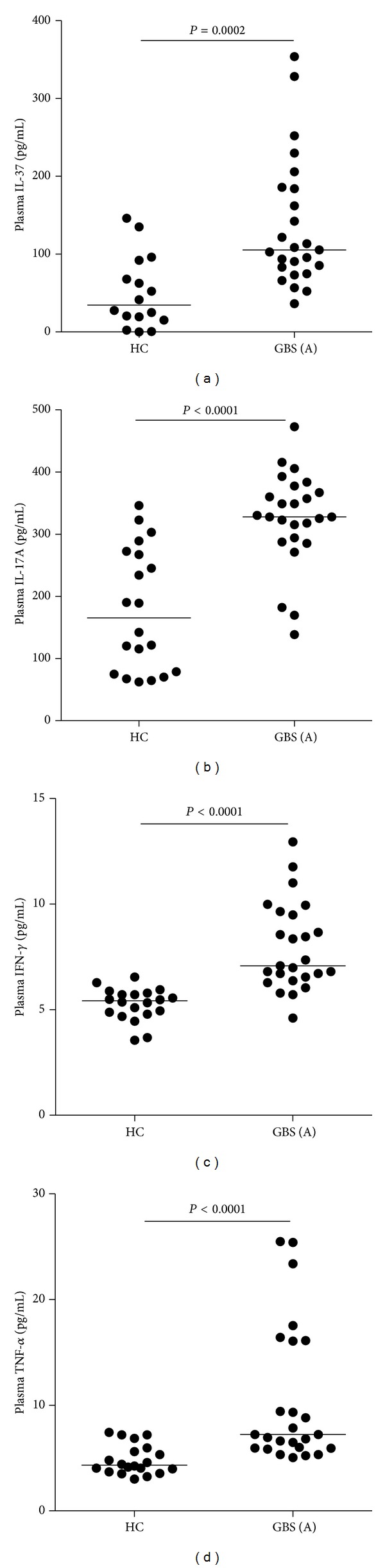
The levels of plasma cytokines in individual subjects. The concentrations of plasma IL-37 (a), IL-17A (b), IFN-*γ* (c), and TNF-*α* (d) in individual patients with new onset GBS (*n* = 25) and the HC (*n* = 20) were determined by ELISA and CBA, respectively. Data shown are the mean values of individual subjects from three separate experiment, and the horizontal lines indicate the median values of individual groups.

Proinflammatory responses have been associated with the pathogenesis of GBS, and IL-37, an anti-inflammatory cytokine, is commonly detected in inflammatory tissues to inhibit excessive inflammation. To investigate the potential role of these cytokines, we examined the concentrations of plasma IL-37, IL-17A, IFN-*γ*, and TNF-*α* in 25 patients with new onset GBS and 20 HC by ELISA and CBA, respectively. We found that the concentrations of plasma IL-37, IL-17A, IFN-*γ*, and TNF-*α* in the patients were significantly higher than those in the HC (Figure 1, *P* = 0.0002 and *P* < 0.0001, resp.).

**Figure 2:**
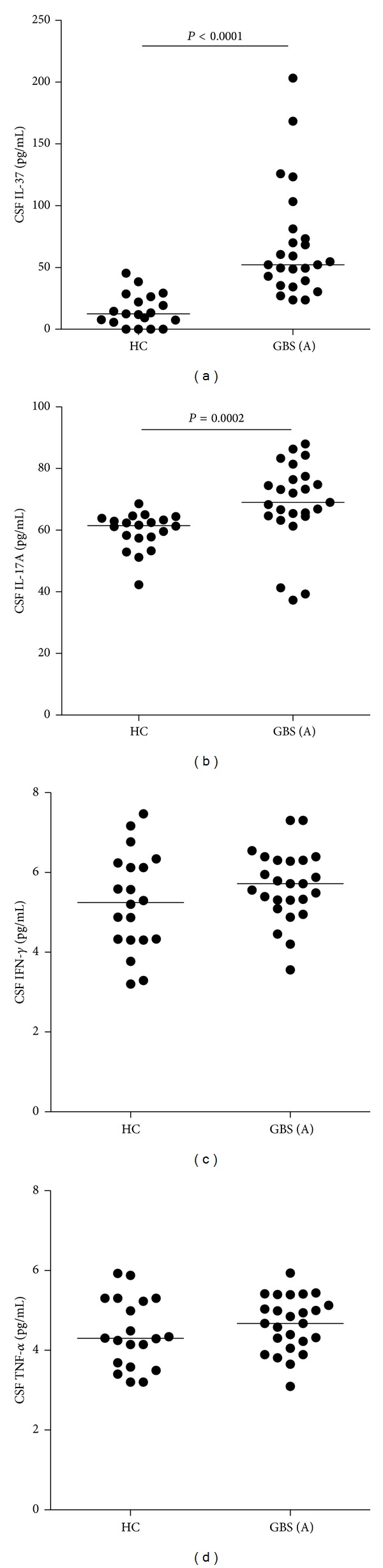
The concentrations of CSF cytokines. The concentrations of CFS IL-37 (a), IL-17A (b), IFN-*γ* (c), and TNF-*α* (d) in individual patients with new onset GBS (*n* = 25) and the HC (*n* = 20) were determined by ELISA and CBA, respectively. Data shown are the mean values of individual subjects from three separate experiment, and the horizontal lines indicate the median values of individual groups.

Characterization of CSF cytokines revealed that the levels of CSF IL-37 and IL-17A were dramatically lower than those of plasma IL-37 and IL-17A in both GBS patients and the HC and that the concentrations of CSF IL-37 and IL-17A in the patients were significantly higher than those in the HC (Figure 2, *P* < 0.0001 and *P* = 0.0002, resp.). In contrast, the levels of CSF IFN-*γ* and TNF-*α* were similar to those of plasma IFN-*γ* and TNF-*α* in both GBS patients and the HC, and there was no significant difference in the levels of CSF IFN-*γ* and TNF-*α* between the GBS patients and the HC in this population. Therefore, significantly higher concentrations of plasma and CSF IL-37 and IL-17A were detected in patients with new onset GBS.

### 3.3. The Levels of Serum and CSF Cytokines Are Correlated with the Values of GDS in GBS Patients

**Figure 3:**
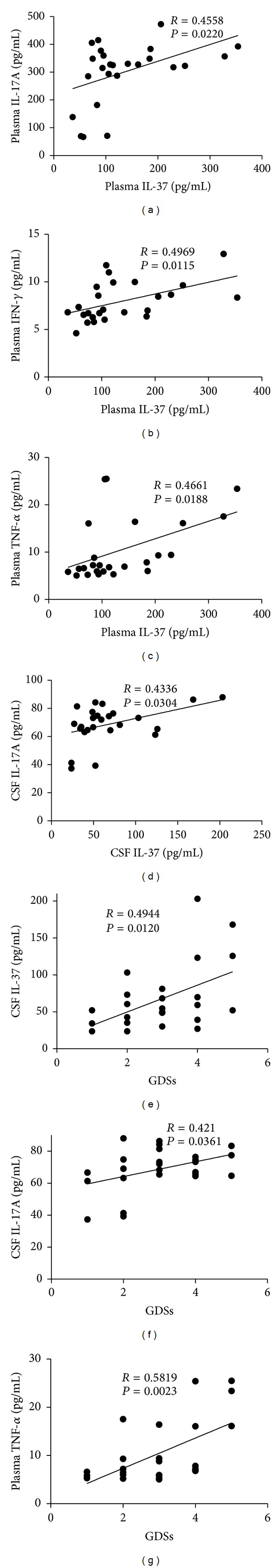
Correlation analyses between the levels of cytokines and the values of GDSs in those patients. The concentrations of plasma IL-37 were positively correlated with the levels of IL-17A (a), IFN-*γ* (b), and TNF-*α* (c) in those patients. The levels of CSF IL-37 were positively correlated with the levels of CFS IL-17A (d) in those patients. The concentrations of CSF IL-37 (e) and IL-17A (f) as well as plasma TNF-*α* were positively correlated with the values of GDSs in those patients.

Next, we analyzed the potential relationships between the levels of cytokines and the values of GDS in those patients. We found that the concentrations of plasma IL-37 were correlated positively with the levels of plasma IL-17A, IFN-*γ*, and TNF-*α* in GBS patients (Figure 3, *R* = 0.4558 and *P* = 0.022; *R* = 0.4969 and *P* = 0.0115; *R* = 0.4661 and *P* = 0.0188, resp.). Similarly, the concentrations of CSF IL-37 were correlated positively with the levels of CSF IL-17A in those patients (*R* = 0.4336 and *P* = 0.0304). Further analysis revealed that the concentrations of CSF IL-37 and IL-17A were positively correlated with the values of GDSs (Figure 3, *R* = 0.4944 and *P* = 0.012; *R* = 0.421 and *P* = 0.0361, resp.). Interestingly, the levels of plasma TNF-*α* were also correlated positively with the values of GDSs in those patients (*R* = 0.5819 and *P* = 0.0023). These data suggest that the abnormal levels of cytokines may be associated with the development of GBS.

### 3.4. Immunoglobulin Therapy Not Only Improves Clinical Symptoms but Also Reduces the Levels of Plasma Cytokines Tested in the Drug Responders but Not in the Drug Nonresponders

**Figure 4:**
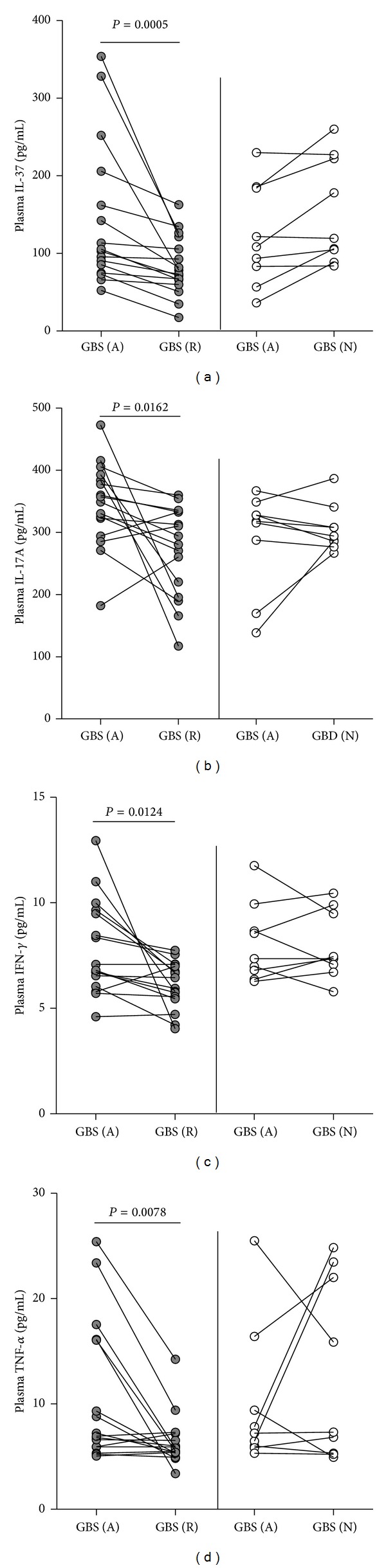
Treatment with IV-Ig significantly reduces the levels of plasma cytokines tested in the drug-responding patients, but not in the drug nonresponders. The concentrations of plasma IL-37 (a), IL-17A (b), IFN-*γ* (c), and TNF-*α* (d) were determined at 4 weeks after the treatment and compared with those before the treatment in 16 therapy responders (grey dots) and nine therapy nonresponders (white dots). Data shown are values of individual patients before and after the treatment. GBS (A): patients at acute stage; GBS (R): drug responders; GBS (N): drug nonresponders.

The IV-Ig therapy has been considered as a standard therapy for patients with active GBS. We treated 25 patients with a course of 5 days of IV-Ig and evaluated the disease scores. Four weeks after the treatment, we found that 16 out of 25 patients were the drug responders, while the remaining nine patients were the drug nonresponders. Analysis of plasma cytokines indicated that the concentrations of plasma IL-37, IL-17A, IFN-*γ*, and TNF-*α* in the drug responders were significantly lower than those before treatment, while there was no significant difference in the levels of plasma IL-37, IL-17A, IFN-*γ*, and TNF-*α* in those drug nonresponders, as compared with those before treatment (Figure 4). Therefore, immunoglobulin treatment reduced the values of GDSs and the levels of plasma cytokines in GBS patients.

## 4. Discussion

GBS is an autoimmune inflammatory demyelinatiing disease affecting the PNS [20, 21]. Previous studies have suggested that proinflammatory Th1 and Th17 are important players in the pathogenesis of GBS [22, 23]. However, it is unclear how these proinflammatory T-cell responses are regulated during the pathogenic process. In this study, we examined the concentrations of plasma and CSF IL-37, IL-17A, IFN-*γ*, and TNF-*α* in 25 patients with new onset GBS and 20 age- and gender-matched the HC. We found that the concentrations of plasma proinflammatory cytokines such as IL-17A, IFN-*γ*, and TNF-*α* in the patients were significantly higher than those in the HC. Similarly, the levels of CSF IL-17A, but not IFN-*γ* and TNF-*α*, in the patients were significantly higher than those in the HC. More importantly, the levels of CSF and plasma IL-17A and plasma TNF-*α* were correlated positively with the GDSs in those patients. These data were consistent with previous findings in humans and rodents [11, 24], supporting the notion that proinflammatory T-cell immunity is crucial for the pathogenesis of GBS. The low concentrations of CSF IFN-*γ* and TNF-*α* in both the patients and the HC may stem from relatively low levels of plasma IFN-*γ* and TNF-*α* detected in this population, except for a few patients. Accordingly, it is unlikely that the low levels of CSF IFN-*γ* and TNF-*α* were from the failure of these cytokines to penetrate into the central nervous system (CNS). Indeed, activated T cells and macrophages can infiltrate into the inflammatory sites in the CNS.

IL-37 has been thought to be an anti-inflammatory cytokine produced by several types of cells [25]. And higher levels of serum IL-37 were detected in patients with autoimmune diseases, such as RA, IBD, and SLE [12, 14, 26, 27]. In this study, we found that the concentrations of plasma and CSF IL-37 were significantly higher in the patients than that in the HC. Furthermore, the levels of plasma IL-37 were correlated positively with the levels of plasma proinflammatory cytokines tested, and the concentrations of CSF IL-37 were correlated positively with the GDSs in those patients. Previous studies have shown that proinflammatory cytokines IL-18, IFN-*γ*, IL-1*β*, and TNF-*α* can increase synthesis of IL-37 in human peripheral blood mononuclear cells [12, 28]. Hence, our data suggest that proinflammatory cytokines may promote anti-inflammatory IL-37 expression to downregulate excessive inflammation during the pathogenic process of GBS. Indeed, IL-37 has been shown to inhibit proinflammatory responses in mice [29–31]. However, we can not completely exclude the possibility that IL-37 may play a pathogenic role in the GBS process in those patients. Given that much higher levels of plasma IL-37 were detected in those patients, it is possible that the penetration of plasma IL-37 into the CNS caused higher levels of CSF IL-37 in those patients. Interestingly, IL-17A can disrupt the blood-brain barrier [32], and we found that the concentrations of CSF IL-17A and IL-37 were correlated positively in those patients. It is also possible that IL-17A may drive the penetrations of IL-37 from plasma to the CNS in GBS patients. Alternatively, proinflammatory cytokines induce IL-37 expression in the CNS that leads to higher levels of CSF IL-37 in those patients. To the best of our knowledge, this was the first study on the levels of plasma and CSF IL-37 in GBS patients. Our findings suggest that the levels of plasma and CSF IL-37 may be used for the evaluation of disease severity in GBS patients. We are interested in further investigating the function of IL-37 during the pathogenic process of GBS.

Currently, patients with GBS are usually treated with IV-Ig. We found that 16 out of 25 patients responded to the standard therapy with reduced GDSs and achieved disease recovery at 4 weeks after treatment. In addition, we found that treatment with IV-Ig significantly reduced the levels of plasma IL-37, IFN-*γ*, TNF-*α*, and IL-17A in those drug responders but not in those drug nonresponders in this population. Given that therapeutic Ig contained multiple antibodies against various antigens, treatment with IV-Ig may neutralize these cytokines, thereby inhibiting the production of cytokines and inflammatory cell function. The reduced levels of plasma IL-37 may also stem from the Ig-mediated inhibition of inflammation, which mitigates the induction of anti-inflammatory IL-37 expression in GBS patients. Therefore, our data clearly demonstrated that treatment with IV-Ig not only alleviated the disease activity but also inhibited inflammation in GBS patients. Conceivably, the levels of plasma cytokines, particularly for IL-17A and IL-37, may be important for evaluating the therapeutic efficacy in GBS patients.

In summary, our data indicated higher levels of plasma IL-37, IFN-*γ*, TNF-*α*, and IL-17A and CSF IL-17A and IL-37 in patients with new onset GBS. The concentrations of plasma and CSF IL-37 and IL-17A were correlated positively with the GDSs in GBS patients. Treatment with the standard therapy of IV-Ig not only significantly alleviated the disease activity, but also reduced the levels of plasma cytokines tested in the drug-responding patients, but not in the drug nonresponding patients. Our findings may provide new insights into the regulation of inflammatory responses during the pathogenic process of GBS and suggest that the levels of plasma and CSF IL-17A and IL-37 may be important for evaluating the disease severity and therapeutic efficacy in GBS patients. We recognized that our study had limitations of small sample size and the lack of a functional study of these cytokines as well as the measurement of CSF cytokines after the treatment. Therefore, further investigations of the potential role of these cytokines in the pathogenesis of GBS in a bigger population are warranted.

## Figures and Tables

**Table 1:** The demographic and clinical characteristics of subjects.

Parameters	Healthy controls	GBS patients
Number	20	25
Age (years)	32 (16–63)	39 (14–56)
Gender (F/M)	8/12	10/15
WBC in CSF (10^6^/L)	0.54 (0.17–1.0)	0.72 (0.21–1.0)
Albumin in CSF (g/L)	0.24 (0.16–0.39)	0.65 (0.49–2.05)*
Albumin in plasma (g/L)	40.53 (35.2–50.67)	42.1 (30.24–53.1)
Qalb × 100	0.59 (0.32–1.11)	1.54 (0.92–6.78)*
Treatment	NA	IV immunoglobulin
WBC (×10^9^/L)	6.7 (4.2–9.3)	8.55 (4.5–26)*
Lymphocytes (×10^9^/L)	2.43 (0.81–3.66)	3.79 (0.94–5.2)
Outcome		
Drug responders	NA	16
Drug nonresponders	NA	9

Data shown are median (range) of each group of subjects. WBC: white blood cells; Qalb: albumin in CSF versus albumin in plasma. (A): acute phase; *P < 0.05 versus the HC.

## Abbreviations

IL: Interleukin
CSF: Cerebrospinal fluid
GBS: Guillain-Barré Syndrome
HC: Healthy controls
GDSs: GBS disability scale scores
BNB: Blood nerve barrier
PNS: Peripheral nervous system
CNS: Central nervous system
IV-Ig: Immunoglobulin
MS: Multiple sclerosis
RA: Rheumatoid arthritis
IBD: Inflammatory bowel disease
SLE: Systemic lupus erythematosus
T1D: Type 1 diabetes
ELISA: Enzyme-linked immunosorbent assay
CBA: Cytometric bead array.

## References

[1] Ho T, Griffin J (1999). Guillain-Barré syndrome. *Current Opinion in Neurology*.

[2] Fish M, Llewelyn G (2008). The Guillain-Barré syndrome. *ACNR*.

[3] Nadkar MY, Bajpai S, Itolikar M (2013). Guillain-Barré syndrome: a common neurological entity with myriad manifestations. *JAPI*.

[4] Ang CW, Jacobs BC, Laman JD (2004). The Guillain-Barré syndrome: a true case of molecular mimicry. *Trends in Immunology*.

[5] van Doorn PA, Ruts L, Jacobs BC (2008). Clinical features, pathogenesis, and treatment of Guillain-Barré syndrome. *The Lancet Neurology*.

[6] Zhu W, Mix E, Nennesmo I, Adem A, Zhu J (2004). Anti-cytokine autoantibodies in experimental autoimmune neuritis in Lewis rats. *Experimental Neurology*.

[7] van der Meché FGA, van Doorn PA, Meulstee J, Jennekens FGI (2001). Diagnostic and classification criteria for the Guillain-Barré syndrome. *European Neurology*.

[8] Nyati KK, Prasad KN, Rizwan A, Verma A, Paliwal VK (2011). TH1 and TH2 response to *Campylobacter jejuni* antigen in Guillain-Barré syndrome. *Archives of Neurology*.

[9] Lu MO, Zhu J (2011). The role of cytokines in Guillain-Barré syndrome. *Journal of Neurology*.

[10] Yu JJ, Gaffen SL (2008). Interleukin-17: a novel inflammatory cytokine that bridges innate and adaptive immunity. *Frontiers in Bioscience*.

[11] Miossec P (2009). IL-17 and Th17 cells in human inflammatory diseases. *Microbes and Infection*.

[12] Boraschi D, Lucchesi D, Hainzl S (2011). IL-37: a new anti-inflammatory cytokine of the IL-1 family. *European Cytokine Network*.

[13] Kumar S, McDonnell PC, Lehr R (2000). Identification and initial characterization of four novel members of the interleukin-1 family. *The Journal of Biological Chemistry*.

[14] Nold MF, Nold-Petry CA, Zepp JA, Palmer BE, Bufler P, Dinarello CA (2010). IL-37 is a fundamental inhibitor of innate immunity. *Nature Immunology*.

[15] Akdis M, Burgler S, Crameri R (2011). Interleukins, from 1 to 37, and interferon-*γ*: receptors, functions, and roles in diseases. *Journal of Allergy and Clinical Immunology*.

[16] Asbury AK, Cornblath DR (1990). Assessment of current diagnostic criteria for Guillain-Barré syndrome. *Annals of Neurology*.

[17] Hughes RAC, Newsom-Davis JM, Perkin GD, Pierce JM (1978). Controlled trial of prednisolone in acute polyneuropathy. *The Lancet*.

[18] Morgan E, Varro R, Sepulveda H (2004). Cytometric bead array: a multiplexed assay platform with applications in various areas of biology. *Clinical Immunology*.

[19] Tárnok A, Hambsch J, Chen R, Varro R (2003). Cytometric bead array to measure six cytokines in twenty-five microliters of serum. *Clinical Chemistry*.

[20] Pithadia AB, Kakadia N (2010). Guillain-Barré syndrome (GBS). *Pharmacological Reports*.

[21] Hughes RAC, Hadden RDM, Gregson NA, Smith KJ (1999). Pathogenesis of Guillain-Barré syndrome. *Journal of Neuroimmunology*.

[22] Zhang HL, Azimullah S, Zheng XY (2012). IFN-*γ* deficiency exacerbates experimental autoimmune neuritis in mice despite a mitigated systemic Th1 immune response. *Journal of Neuroimmunology*.

[23] Li S, Yu M, Li H, Zhang H, Jiang Y (2012). IL-17 and IL-22 in cerebrospinal fluid and plasma are elevated in Guillain-Barré syndrome. *Mediators of Inflammation*.

[24] Lambracht-Washington D, Wolfe GI (2011). Cytokines in Guillain-Barré syndrome: a lesson in time. *Archives of Neurology*.

[25] Sharma S, Kulk N, Nold MF (2008). The IL-1 family member 7b translocates to the nucleus and down-regulates proinflammatory cytokines. *Journal of Immunology*.

[26] Banchereau J, Pascual V, O'Garra A (2012). From IL-2 to IL-37: the expanding spectrum of anti-inflammatory cytokines. *Nature Immunology*.

[27] Song L, Qiu F, Fan Y (2013). Glucocorticoid regulates interleukin-37 in systemic lupus erythematosus. *Journal of Clinical Immunology*.

[28] Tete S, Tripodi D, Rosati M (2012). IL-37 (IL-1F7) the newest anti-inflammatory cytokine which suppresses immune responses and inflammation. *International Journal of Immunopathology and Pharmacology*.

[29] McNamee EN, Masterson JC, Jedlicka P (2011). Interleukin 37 expression protects mice from colitis. *Proceedings of the National Academy of Sciences of the United States of America*.

[30] Bulau AM, Fink M, Maucksch C (2011). In vivo expression of interleukin-37 reduces local and systemic inflammation in concanavalin A-induced hepatitis. *TheScientificWorldJOURNAL*.

[31] Sakai N, van Sweringen HL, Belizaire RM (2012). IL-37 reduces liver inflammatory injury via effects on hepatocytes and non-parenchymal cells. *Journal of Gastroenterology and Hepatology*.

[32] Kebir H, Kreymborg K, Ifergan I (2007). Human TH17 lymphocytes promote blood-brain barrier disruption and central nervous system inflammation. *Nature Medicine*.

